# Evaluating the Diagnostic Accuracy of an Artificial Intelligence Tool for Pulmonary Abnormalities in Chest Radiographs: A Retrospective Study

**DOI:** 10.7759/cureus.92690

**Published:** 2025-09-19

**Authors:** Mauro Gobira, Marina D Moreira do Amaral, Osvaldo Landi Júnior, Gladstone Mattar, Marcelo Itiro Takano, Eric P De Andrade

**Affiliations:** 1 Ophthalmology, Vision Institute (IPEPO), São Paulo, BRA; 2 Ophthalmology, Hospital do Servidor Público Estadual de São Paulo, São Paulo, BRA; 3 Radiology, Fundação Instituto de Pesquisa e Estudo de Diagnóstico por Imagem, São Paulo, BRA; 4 Radiology, Hospital do Servidor Público Estadual de São Paulo, São Paulo, BRA; 5 Ophthalmology, Universidade Federal de São Paulo, São Paulo, BRA

**Keywords:** artificial intelligence, chest radiography, computer-assisted diagnosis, pulmonary diseases, telemedicine

## Abstract

Introduction

A retrospective, in-clinic study was conducted to validate the diagnostic performance of the Oxipit ChestEye™ AI tool in interpreting chest radiographs and to evaluate its impact on clinical outcomes and healthcare system efficiency. The performance of the AI model was compared with that of radiologists.

Methods

Chest radiographs from 1,470 patients, obtained from a public healthcare institution, were analyzed for nine pulmonary conditions: consolidation, heart enlargement, interstitial markers, linear atelectasis, no cardiopulmonary findings, nodule, pleural effusion, pneumothorax, and tuberculosis. Diagnostic accuracy, sensitivity, specificity, positive predictive value (PPV), negative predictive value (NPV), and F1 scores were calculated for Oxipit ChestEye™ and compared with radiologists' diagnoses, which served as the reference standard.

Results

The AI tool demonstrated accuracy ranging from 0.79 to 1.00 across the nine conditions evaluated. Sensitivity ranged from 0.25 to 1.00, and specificity ranged from 0.65 to 1.00. In differentiating between "Normal" and "Altered" categories, the AI model achieved an accuracy of 0.78. The F1 score for "Normal" was 0.86, whereas for "Altered," it was 0.52.

Conclusions

Oxipit ChestEye™ demonstrated high accuracy and efficiency in detecting and triaging pulmonary abnormalities in chest radiographs. While it is a useful tool to supplement the work of the radiologists, helping to reduce diagnostic time and improve patient care, it should not replace human radiologists.

## Introduction

Lung diseases remain one of the leading global health challenges, accounting for approximately one-sixth of all deaths worldwide [1]. Despite the advances in diagnostics and therapeutics, the global burden of respiratory conditions remains significant and continues to impose profound socioeconomic burdens, including increased hospitalizations and reduced workforce productivity [1]. Artificial intelligence (AI) has emerged as a potential tool in radiology, aiming to improve diagnostic accuracy, optimize workflow efficiency, and reduce reporting delays [2,3]. A recent census by the Royal College of Radiologists reported that over 745,000 patients in the United Kingdom experience delays exceeding four weeks for radiological report delivery, emphasizing the need for scalable image interpretation solutions [2,3].

A commercially available deep learning model for chest radiograph interpretation, ChestEye (Oxipit Ltd., Vilnius, Lithuania), has been developed to detect multiple thoracic abnormalities. While technical details regarding its architecture, training, and dataset composition are not publicly disclosed, previous studies have reported promising diagnostic performance [4]. We selected ChestEye because it was available at our institution, had prior peer-reviewed validation reports, and was already in commercial use within our region, facilitating real-world implementation. We conducted an exploratory, retrospective validation study evaluating the tool’s diagnostic accuracy in chest radiographs from a public healthcare institution and comparing its performance with that of board-certified thoracic radiologists to gauge its feasibility as a clinical support system in real-world conditions.

## Materials and methods

Study design and ethical approval

This retrospective observational study compared the diagnostic performance of a commercially available AI tool, ChestEye, with that of board-certified thoracic radiologists in interpreting chest radiographs for nine predefined pulmonary conditions. All radiological reports used as the reference standard were issued by thoracic imaging specialists. The study received ethical approval from the Research Ethics Committee of the Hospital do Servidor Público Estadual (IAMSPE), São Paulo, Brazil.

Data collection

Chest radiographs were retrospectively retrieved for adult patients evaluated between June 1 and August 30, 2024. The analysis focused on the following diagnostic categories: consolidation, cardiomegaly, interstitial markings, linear atelectasis, absence of cardiopulmonary findings, nodules, pleural effusion, pneumothorax, and tuberculosis. Inclusion criteria required standard posteroanterior (PA) or anteroposterior (AP) chest X-rays with adequate image quality and associated diagnostic reports. All exams were single-view PA or AP; lateral views were not included. Exclusion criteria included technically inadequate images, missing clinical data, or inability to provide consent when applicable.

**Figure 1:**
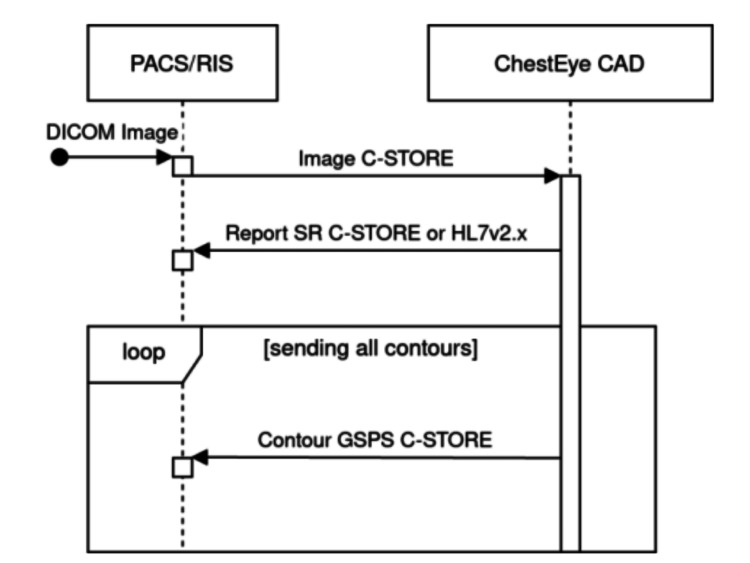
Example data flow chart of the report delivery module Image credit: Eric Pinheiro de Andrade

The reference standard dataset was derived from radiology reports using a customized natural language processing framework based on large language models and tailored regular expressions, developed in collaboration with thoracic radiologists to ensure high-fidelity extraction of diagnostic terms. To enhance reliability, a random subset of reports was manually cross-checked by two thoracic radiologists, with discrepancies resolved by consensus. Radiographs were transferred from the institutional picture archiving and communication system (PACS) to the ChestEye platform using secure DICOM transmission (Figure 1).

Statistical analysis

Due to the exploratory nature of the study, no a priori sample size calculation was performed. All eligible radiographs within the defined period were included to enhance representativeness. The primary outcome was the diagnostic accuracy of the AI tool for each condition. Sensitivity, specificity, positive predictive value (PPV), negative predictive value (NPV), and area under the receiver operating characteristic curve (AUC) were calculated. Confidence intervals (CIs) were computed for all metrics using the DataTAB statistical platform (DataTAB e.U., Graz, Austria) [5].

The AI produced binary outputs per category, compared against the final signed radiologist report as the reference. Reports were single-read; no consensus re-reads were performed. When addenda existed, the latest report defined the label. Discrepancies were qualitatively reviewed to characterize error patterns. No external benchmark datasets were used.

## Results

A total of 1,470 patients were included in the final analysis of chest radiographs: 1,457 individuals from ambulatory care, 10 from inpatient settings, and three from the emergency department. The performance of the AI model, ChestEye, varied across the nine evaluated diagnostic categories (Table 1).

**Table 1:** Diagnostic accuracy of Oxipit.ai in identifying pulmonary abnormalities from chest radiographs CI: confidence interval

Condition	Accuracy	Sensitivity (95% CI)	Specificity (95% CI)	True positives	False positives	True negatives	False negatives
Consolidation	0.96	0.50 (0.27–0.73)	0.97 (0.96–0.98)	7	45	1411	7
Enlarged heart	0.93	0.25 (0.18–0.34)	0.99 (0.98–0.99)	29	20	1334	87
Interstitial markings	0.95	0.31 (0.21–0.44)	0.97 (0.96–0.98)	19	38	1371	42
Linear atelectasis	0.99	0.13 (0.04–0.38)	1.00 (0.99–1.00)	2	5	1450	13
No cardiopulmonary findings	0.79	0.83 (0.80–0.85)	0.65 (0.59–0.70)	996	93	170	211
Nodules	0.94	0.55 (0.42–0.67)	0.96 (0.95–0.98)	36	58	1347	29
Pleural effusion	0.98	0.65 (0.44–0.82)	0.99 (0.99–1.00)	13	16	1434	7
Pneumothorax	1	1.00 (0.025–1.00)	1.00 (0.99–1.00)	1	0	1469	0
Tuberculosis	1	1.00 (0.48–1.00)	1.00 (0.99–1.00)	5	0	1465	0

For the detection of consolidation, the model demonstrated an accuracy of 0.96, with a sensitivity of 0.50 (95%: 0.27-0.73) and a specificity of 0.97 (95% CI: 0.96-0.98). Of the 14 evaluated cases, seven were true positives and seven were false negatives. Additionally, 45 false positives and 1,411 true negatives were recorded. In the evaluation of cardiomegaly, the model achieved an accuracy of 0.93, with a sensitivity of 0.25 (95% CI: 0.18-0.34) and a specificity of 0.99 (95% CI: 0.98-0.99). Among 116 cases, 29 were true positives and 1,334 were true negatives, while 87 false negatives and 20 false positives were noted.

For interstitial markings, the model demonstrated an accuracy of 0.95, a sensitivity of 0.31 (95% CI: 0.21-0.44), and a specificity of 0.97 (95% CI: 0.96-0.98). Of the 61 relevant cases, 19 were true positives and 1,371 were true negatives, with 38 false positives and 42 false negatives. In the linear atelectasis category, the model achieved an accuracy of 0.99, sensitivity of 0.13 (95% CI: 0.04-0.38), and specificity of 1.00 (95% CI: 0.99-1.00). Among 15 cases, two were true positives, 13 were true negatives, five were false positives, and 13 false negatives were reported.

For the “No Cardiopulmonary Findings” category, which encompassed the largest number of cases (n = 1,207), the model demonstrated an accuracy of 0.79, sensitivity of 0.83 (95% CI: 0.80-0.85), and specificity of 0.65 (95% CI: 0.59-0.70). The model identified 996 true positives and 170 true negatives, while generating 211 false negatives and 93 false positives. In the identification of nodules, the model achieved an accuracy of 0.94, with a sensitivity of 0.55 (95% CI: 0.42-0.67) and specificity of 0.96 (95% CI: 0.95-0.98). Among 65 cases, 36 were true positives and 1,347 were true negatives. The model generated 58 false positives and 29 false negatives.

For pleural effusion, the model attained an accuracy of 0.98, with a sensitivity of 0.65 (95% CI: 0.44-0.82) and a specificity of 0.99 (95% CI: 0.99-1.00). Out of 20 cases, 13 were true positives and 1,434 were true negatives, with 16 false positives and seven false negatives. In the detection of pneumothorax, the model demonstrated perfect performance with an accuracy of 1.00, sensitivity of 1.00 (95% CI: 0.20-1.00), and specificity of 1.00 (95% CI: 0.99-1.00). The single case in this category was correctly identified as a true positive, with 1,469 true negatives and no false classifications.

**Figure 2:**
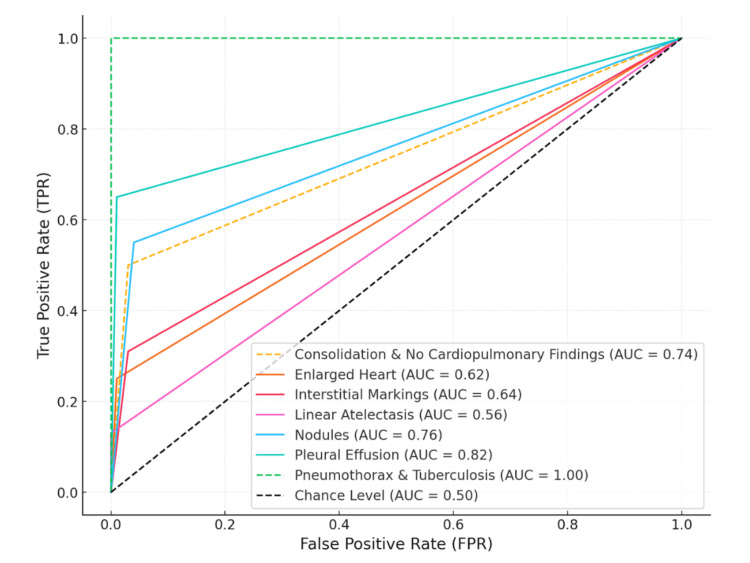
ROC curves for diagnostic categories ROC: receiver operating characteristic; AUC: area under the ROC curve

Similarly, for tuberculosis, the model also reached an accuracy of 1.00, with a perfect sensitivity of 1.00 (95% CI: not applicable) and a specificity of 1.00 (95% CI: not applicable). All five cases were correctly identified as true positives, and the remaining 1,465 images were true negatives, with no false positives or false negatives. Finally, in the binary classification task, differentiating between “Normal” and “Altered” images, the model achieved an overall accuracy of 0.78 for both categories. For the “Normal” group, sensitivity was 0.80, specificity was 0.67, and the F1 score was 0.86. For the “Altered” group, sensitivity was 0.67, specificity was 0.80, and the F1 score was 0.52. The receiver operating characteristic (ROC) curve illustrating model performance across categories is shown in Figure 2.

## Discussion

AI has demonstrated considerable progress in the interpretation of chest radiographs, contributing to enhanced diagnostic accuracy and improved clinical workflows across a wide range of thoracic pathologies [6-12]. In this study, the AI model ChestEye was evaluated using a retrospective dataset from a public healthcare institution, where the average radiological report turnaround time ranged from 48 hours to seven days. In contrast, the AI system operates in near real-time, offering immediate assessments that may enable earlier clinical decisions and potentially influence patient outcomes [6-12].

Unlike most prospective studies conducted in primary care settings, the present analysis provides insight into the performance of the model under real-world conditions in a tertiary care environment. The results showed variable diagnostic performance across nine categories, with accuracy ranging from 0.79 to 1.00, sensitivity from 0.25 to 1.00, and specificity from 0.65 to 1.00. These findings contrast with those of Catalina et al. [12], who reported an average accuracy of 0.95 (95% CI: 0.92-0.98), sensitivity of 0.48 (95% CI: 0.30-0.66), and specificity of 0.98 (95% CI: 0.97-0.99) in a prospective analysis of 278 radiographs. In another study, Codlin et al. [11] reported lower accuracy values (0.73; 95% CI: 0.69-0.77) for tuberculosis detection, highlighting variability across models and contexts.

Beyond this specific platform, studies evaluating other convolutional neural networks, such as DenseNet121, have demonstrated AUC values up to 94% for pneumothorax and pulmonary edema detection [8]. The Rayvolve model, for instance, was shown to increase diagnostic AUC by 15.94% while reducing interpretation time by more than 35% [8]. Additionally, automated cardiothoracic ratio (CTR) measurements have shown high concordance with manual readings, with systematic reviews reporting pooled AUC values of 0.959, supporting the application of AI in assessing cardiomegaly [9].

Despite these advances, significant limitations persist. AI models typically underperform when identifying small (less than 1 cm) or peripherally located nodules, particularly when these are obscured by overlapping anatomical structures [10]. Although many tools report high AUCs in internal validations, external radiologist-led evaluations have identified inconsistencies in segmentation performance, underscoring the importance of rigorous external validation [10].

From a workflow perspective, AI in chest radiography can be integrated as a triage tool or as a first-line screener [10]. Triage applications appear most feasible, as high specificity and negative predictive value allow prioritization of abnormal studies and reduction of reporting delays [10]. However, the presence of false negatives limits its safety in this role, as missed findings may delay critical diagnoses. First-line screening remains premature without stronger external validation. Given its performance profile, ChestEye may be better positioned for triage under close radiologist supervision rather than standalone interpretation.

Limitations

A significant limitation of the present study is the absence of publicly available benchmark datasets such as ChestX-ray14 [13], which may limit the reproducibility and generalizability of the findings. Additionally, the dataset used lacked geographic and ethnic diversity, which may constrain its applicability across broader populations. Furthermore, while this analysis compared AI predictions to radiologist interpretations, it did not assess the clinical impact of integrating AI into diagnostic workflows. Future research should address how such tools affect radiologists' performance, confidence, report turnaround times, and ultimately, patient outcomes. Prospective studies incorporating clinical integration endpoints - such as changes in diagnostic decisions and accuracy when used as a second reader - are essential to establish real-world clinical utility.

In summary, the findings of this study reinforce the potential role of AI in supporting chest radiograph interpretation, particularly in settings with limited resources. However, the variability in sensitivity across diagnostic categories highlights the need for further model calibration. Future studies should prioritize prospective, large-scale validation across diverse patient populations and clinical environments, with emphasis on workflow integration and measurable clinical impact.

## Conclusions

The AI model ChestEye demonstrated high diagnostic accuracy and specificity in detecting multiple thoracic conditions from chest radiographs, with notably accurate identification of pneumothorax and tuberculosis. However, its varying sensitivity in detecting nodules, cardiomegaly, and interstitial markings indicates areas that require further optimization. The model’s capability to generate results in near real-time presents a potential advantage for minimizing diagnostic delays in public healthcare settings. These findings highlight the importance of conducting further validation studies using larger and more diverse datasets to address current limitations, particularly regarding sensitivity variability. Future research should also focus on evaluating AI integration into clinical workflows and assessing its longitudinal impact on diagnostic performance and patient outcomes.
